# Ethanol-Induced Deposition of a Passivation Layer to Enhance the Water Resistance and Luminescent Properties of K_2_TiF_6_: Mn^4+^ Phosphors

**DOI:** 10.3390/ma19101925

**Published:** 2026-05-08

**Authors:** Haoyu Yang, Xinhua Li, Yongyi Gu, Sifei Liu, Xueyu Liu, Xinrong Tang, Haiyan Xu

**Affiliations:** 1 School of Materials and Chemical Engineering, Anhui Jianzhu University, Hefei 230601, China; 2 School of Mathematics & Physics, Anhui Jianzhu University, Hefei 230601, China; 3 Anhui Research Center of Generic Technology in New Display Industry, Hefei 230601, China

**Keywords:** K_2_TiF_6_:Mn^4+^, red phosphor, moisture resistance, surface passivation, coating modification

## Abstract

Poor moisture resistance represents a critical bottleneck restricting the practical application of K_2_TiF_6_:Mn^4+^ phosphors in white light-emitting diodes. In this work, phosphorous acid was employed as a reducing agent to eliminate surface Mn^4+^ species and passivate the phosphor surface, followed by ethanol-induced surface deposition. This approach successfully constructed a thick K_2_TiF_6_ matrix coating layer on the phosphor particles. After 6 h of water immersion, the treated phosphors maintained 88.1% of their initial fluorescence intensity; even after boiling treatment, the internal quantum yield still reached as high as 76.8%. WLED devices encapsulated with the modified phosphors exhibited outstanding stability during continuous operation for 432 h under high-temperature and high-humidity conditions. This effective surface modification strategy significantly broadens the application prospects of Mn^4+^-doped fluoride red phosphors in WLEDs.

## 1. Introduction

Benefiting from high luminous efficiency, energy-saving performance, durability, and environmental friendliness, white light-emitting diodes (WLEDs) have over time become the primary choice for lighting needs in human life [1,2,3]. Commercial WLED devices are typically fabricated by combining red and yellow phosphors with blue LED chips, which effectively improves spectral continuity and boosts the color rendering index [4,5,6]. Among various red phosphors, Mn^4+^-activated fluoride phosphors feature narrow-band red emission under blue light excitation, making them ideal candidates for the red component in high-performance WLEDs [7,8,9,10]. Nevertheless, such fluoride phosphors are highly vulnerable to moisture-induced hydrolysis, which leads to severe luminescence degradation and prevents long-term stable operation in high-temperature and high-humidity environments.

To address this issue, extensive efforts have been devoted to enhancing the moisture resistance of Mn^4+^-doped fluoride phosphors, mainly by constructing a protective waterproof layer on the particle surface to isolate environmental moisture. According to previous reports, surface protective layers can be generally divided into inorganic and organic types. Representative inorganic materials include aluminum oxide and silicon dioxide [11,12]. Although such inorganic coatings provide certain waterproofing effects, it remains difficult to achieve complete, uniform, and defect-free coverage. Moreover, abundant crystal defects are easily introduced at the coating–phosphor interface, which inevitably deteriorates the photoluminescence quantum yield of the phosphors. Consequently, research attention has shifted to organic coating materials owing to their rich varieties, high optical transparency, and good flexibility. Typical organic modifiers include alkyl phosphate, octadecyltrimethoxysilane, and oleic acid, which have been widely used for phosphor surface modification [13,14,15]. However, these organic coatings suffer from complicated preparation procedures and insufficient stability under harsh conditions such as high temperatures and ultraviolet irradiation, limiting their long-term practical applications. In view of these limitations, the reduction passivation strategy has been proposed to construct a core–shell structure on the phosphor surface. The mechanism relies on the redox reaction between a suitable reducing agent and surface-exposed Mn^4+^ ions, generating a low-Mn^4+^-content matrix shell that acts as a moisture barrier and preserves luminescence stability. Citric acid is a representative reducing agent used in such treatments [16,17]. Reduction passivation can significantly improve moisture resistance with negligible influence on optical performance. Nevertheless, the passivation shell formed by conventional reduction treatment is usually ultrathin, so residual Mn^4+^ ions in the outer layer may still undergo hydrolysis under extreme high-temperature and high-humidity service conditions.

Against this background, an ethanol-induced optimized reduction passivation strategy is proposed in this work. Specifically, phosphorous acid (H_3_PO_3_) is first used as a reducing agent to realize surface passivation, and ethanol is then introduced to induce the deposition of a thickened K_2_TiF_6_ (KTF) matrix layer, aiming to construct a compact and robust core–shell structure. A series of characterizations and measurements will be performed to systematically investigate the morphology, structure, photoluminescence properties, water resistance, and high-temperature/high-humidity stability of the modified K_2_TiF_6_:Mn^4+^ phosphors, as well as the operational reliability of corresponding WLED devices. It is expected that modified phosphor can maintain excellent luminescence intensity after water immersion, and the WLED devices packaged with the optimized phosphor will exhibit significantly enhanced environmental stability and longer service life under harsh conditions. This study is expected to provide an effective surface modification route for Mn^4+^-doped fluoride red phosphors and promote their practical applications in high-stability WLEDs.

## 2. Materials and Experimental Methods

### 2.1. Preparation of Phosphors

All reagents used in this experiment were provided by Shanghai MacLinn Co., Ltd. (Shanghai, China). The experiment employed a two-step cation exchange method to prepare the required K_2_TiF_6_:Mn^4+^ phosphor [18]. First, 0.9 g of KMnO_4_ (99%) and 15 g of KHF_2_ (95%) were added to a polytetrafluoroethylene beaker, then 30 mL of HF (49 wt%) was added and the mixture was stirred thoroughly for 30 min under an ice water bath (0 °C). Subsequently, H_2_O_2_ (30 wt%) was slowly added dropwise at a rate of one drop every 5 min until a yellow precipitate formed. The mixture was rinsed multiple times with HF and methanol (99.5%) before the precipitate was collected. The product was dried at 70 °C for 3 h to obtain the K_2_MnF_6_ precursor. K_2_TiF_6_:Mn^4+^ phosphor was prepared by cation exchange method. 3 g of K_2_TiF_6_ (99.5%) was weighed and dissolved in a small amount of HF, stirred thoroughly. Then 0.2 g of the K_2_MnF_6_ precursor was added, stirred for 10 min, followed by multiple washes with a small amount of ethanol (99%). Centrifugal separation yielded a yellow precipitate, which was dried at 70 °C for 3 h. The resulting phosphor was designated as KTFM. 0.5 g of H_3_PO_3_ (99.5%) was taken and dissolved completely in 10 mL of deionized water to form an H_3_PO_3_ solution. Then 0.5 g of KTFM was added to the solution and stirred continuously for 30 min. After washing with ethanol in small amounts multiple times, the mixture was centrifuged to collect the precipitate and dried in a 70 °C oven for 3 h. The resulting phosphor was designated as KTFM-P. In the KTFM-P sample, 20 mL ethanol was added and left undisturbed for 10 min. The mixture was then centrifuged and filtered to collect the yellow precipitate, which was dried in a 70 °C oven for 3 h. The resulting phosphor was designated as KTFM-P@KTF.

### 2.2. WLEDs Packaging and Aging

The prepared fluoride phosphor was mixed with Y_3_Al_5_O_12_:Ce^3+^ yellow phosphor (YAG), then coated onto blue LED chips along with a small amount of epoxy resin, followed by uniform encapsulation using a curing agent. The encapsulated devices were dried in an oven at 70 °C for 3 h to obtain the desired WLED devices. The mass ratio of YAG:KTFM:epoxy resin was 1:5:20. The photoelectric performance of the prepared WLEDs at room temperature was measured under 4 V and 100 mA driving current. Long-term optical stability of the WLEDs was evaluated through aging tests conducted at high temperature (85 °C) and high humidity (85%) for 432 h.

### 2.3. Characterizations

The crystal structure of the phosphor was analyzed using Cu-Kα radiation from an X-ray diffractometer (XRD, Rigaku Smart Lab 9 kw, Rigaku Corporation, Tokyo, Japan). Morphological analysis was performed using a scanning electron microscope (SEM, ZEISS Sigma 300, Carl Zeiss AG, Oberkochen, Germany). In addition, surface element distribution of phosphors was determined by energy-dispersive X-ray spectrometer (EDS, ZEISS Sigma 300, Carl Zeiss AG, Oberkochen, Germany). Morphological characteristics of phosphors were characterized by transmission electron microscopy (TEM, FEI Tecnai F20, FEI, Hillsboro, OR, USA). Surface element composition was measured using X-ray photoelectron spectroscopy (XPS, Thermo ESCALAB 250Xi, Thermo Fisher Scientific, Waltham, MA, USA). Photoluminescence (PL) spectra and luminescence quantum yield (IQY) were measured with the Edinburgh FLS-1000 (Edinburgh Instruments, Livingston, UK) fluorescence spectrometer. Photoelectric properties of fabricated devices were evaluated using an LED photoelectric analyzer (PCE300B, EVERUPING OPTICS, Hangzhou, China).

## 3. Results and Discussion

### 3.1. Structure, Morphology and Composition Analysis

**Figure 1:**
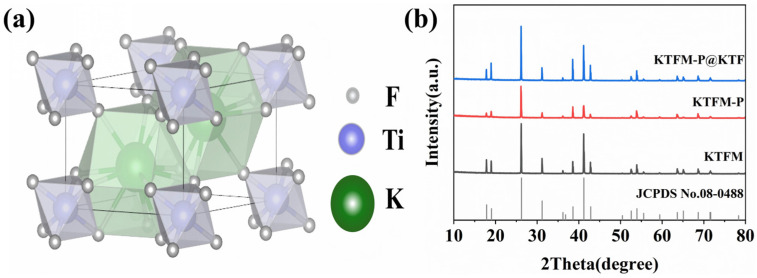
(**a**) Crystal structure of K_2_TiF_6_; (**b**) XRD patterns of KTFM, KTFM-P, and KTFM-P@KTF samples.

K_2_TiF_6_ crystallizes in a trigonal crystal system with the space group P-3m1 (164). In the structure, Ti^4+^ ions are located at the central site and coordinate with six F^−^ ions to form a regular [TiF_6_]^2−^ octahedral configuration. The structural model of K_2_TiF_6_ is illustrated in Figure 1a. K_2_TiF_6_:Mn^4+^(KTFM) phosphors and the corresponding modified samples were synthesized based on the K_2_TiF_6_ host lattice. Figure 1b displays the XRD patterns of KTFM, KTFM-P, and KTFM-P@KTF phosphors. All diffraction peaks are in good agreement with the standard card of K_2_TiF_6_ (JCPDS No. 08-0488) [19], and no obvious impurity peaks are detected. These results confirm that the phosphorous acid reduction and ethanol-induced deposition treatments do not change the phase structure of KTFM phosphor.

**Figure 2:**
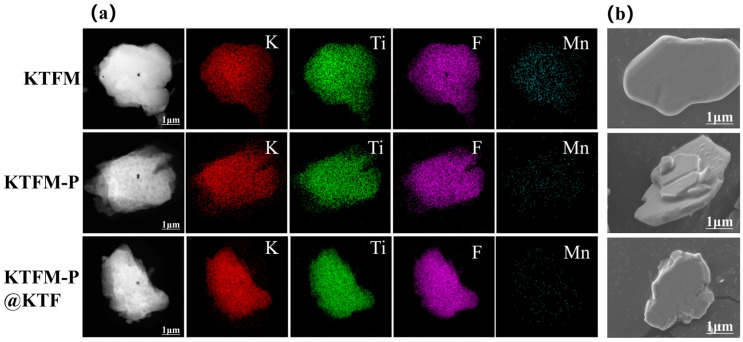
(**a**) EDS mapping and (**b**) SEM images of elemental compositions for KTFM, KTFM-P, and KTFM-P@KTF samples.

As shown in the EDS mapping surface scan of Figure 2a, the K, Ti, and F element signals on the surfaces of KTFM, KTFM-P, and KTFM-P@KTF phosphors exhibit minimal differences, with the most significant variation residing in the Mn signal intensity. Compared to KTFM, KTFM-P shows a markedly weaker Mn signal intensity, which is likely attributed to the redox reaction between phosphorous acid and the [MnF_6_]^2−^ groups on the phosphor surface, leading to the reduction of Mn^4+^ valence state. This reductive passivation successfully constructs a K_2_TiF_6_ (KTF) core–shell structure on the phosphor surface [13]. However, the KTF matrix shell layer formed by this core–shell structure is relatively thin and remains susceptible to hydrolysis under prolonged humid conditions. The undetectable Mn signal intensity in KTFM-P@KTF samples indicates that ethanol-induced deposition results in a thicker KTF matrix shell layer on the phosphor surface, which can effectively block moisture ingress and protect the internal luminescence centers of the phosphor.

Under SEM observation, KTFM presents flaky irregular polygons with smooth surfaces. As illustrated in Figure 2b, the KTFM-P surface shows slight roughness with minor irregularities and high adhesion, where some particles undergo violent fragmentation under high-speed agitation [20]. The increased surface roughness of KTFM-P may be caused by phosphate erosion; similar roughness variations are observed when phosphors are treated with other reducing agents. The adhesion phenomenon in phosphors is likely derived from the inherent viscosity of phosphate compounds. Ethanol-induced treatment significantly reduces the adhesion of KTFM-P@KTF samples, with greatly reduced surface irregularities and restored flatness. This effect is attributed to the ethanol-induced deposition of K_2_TiF_6_ solids, which can effectively fill the surface voids of the phosphors.

To further analyze elemental variations, XPS analysis was conducted on each sample. By integrating the main elemental peaks, the elemental composition ratios of the phosphor and its modified surface were calculated, as shown in Table 1. The table indicates that the C and O contents in KTFM likely originate from atmospheric CO_2_. Comparative analysis between KTFM-P and KTFM-P@KTF samples reveals higher concentrations of F, K, and Ti in the latter: KTFM-P@KTF phosphors exhibit 12.53% K, 45.22% F, and 5.32% Ti, respectively, which are significantly higher than those of KTFM-P (10.47% K, 40.62% F, and 4.64% Ti). This change confirms the formation of a thicker KTF matrix layer on the surface of KTFM-P@KTF samples.

**Figure 3:**
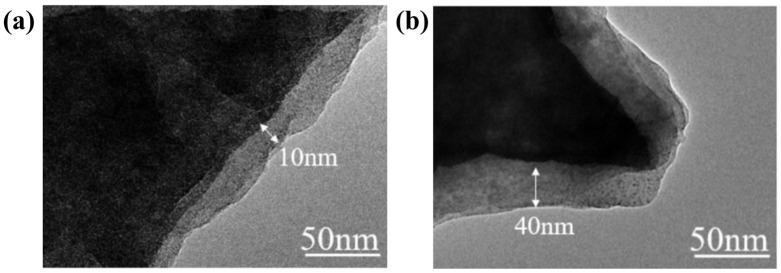
(**a**) TEM image of KTFM-P sample; (**b**) TEM image of KTFM-P@KTF sample.

To further validate the aforementioned hypothesis, TEM was utilized for structural characterization. Observations of the core–shell structure on the phosphor surface at room temperature revealed distinct morphological features. As presented in Figure 3a, the KTFM-P phosphor possessed a thin matrix shell layer of only 10 nm, which is believed to originate from phosphite-induced reduction passivation, leading to the formation of a Mn^4+^-deficient layer within the K_2_TiF_6_ host. Figure 3b shows that the matrix shell layer constructed on KTFM-P@KTF reached a thickness of approximately 40 nm, providing direct evidence that the ethanol-assisted optimized reduction strategy facilitates the formation of a thicker water-resistant coating on the phosphor surface. The reinforced K_2_TiF_6_ protective layer substantially enhances the stability of the phosphor under harsh conditions, delivering reliable shielding for the embedded phosphor particles.

### 3.2. Moisture Resistance Analysis

**Figure 4:**
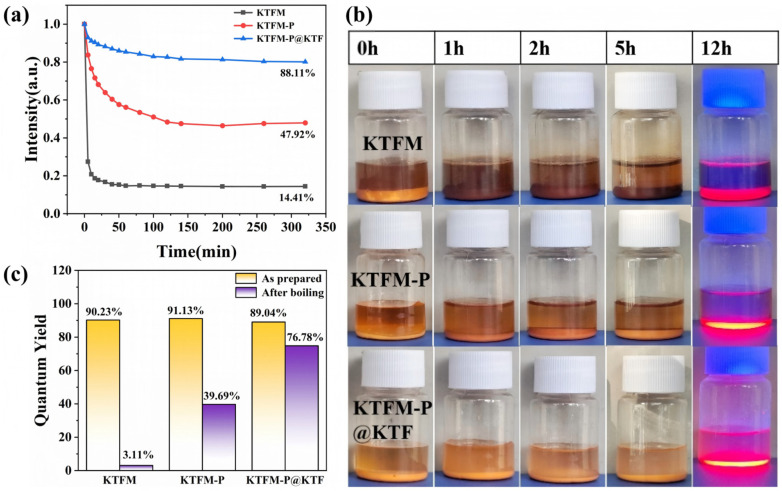
(**a**) PL intensities of KTFM, KTFM-P, and KTFM-P@KTF as a function of water immersion time; (**b**) time-dependent photographs of the samples during water immersion; (**c**) IQY histograms before and after exposure to boiling water.

To evaluate the water resistance performance of modified samples, KTFM, KTFM-P, and KTFM-P@KTF phosphors were immersed in deionized water at room temperature for observation over different time durations. Figure 4a displayed real-time PL intensity changes measured via an integrating sphere (normalized to initial intensity of 100%). After 1 h immersion, KTFM’s luminescence intensity plummeted to 14.4%, while KTFM-P exhibited gradual decay to 47.9% within 6 h. During the same 6 h period, KTFM-P@KTF showed only 11.9% intensity reduction. As illustrated in Figure 4b, KTFM samples demonstrated significant hydrolysis after approximately 1 h immersion, with powder color transitioning from yellow to dark brown. KTFM-P also exhibited color deepening, whereas KTFM-P@KTF maintained stable coloration. After 12 h immersion, localized color darkening occurred in KTFM-P samples, though the degree remained substantially lower than KTFM due to protective effects of the coating formed by surface reduction passivation. Notably, KTFM-P@KTF samples retained their original yellow hue and emitted intense red light under blue light excitation, confirming that the thick KTF coating played critical protective roles. Irradiation with 356 nm UV lamps revealed significantly lower luminescence intensity in KTFM samples compared to KTFM-P and KTFM-P@KTF, with KTFM-P@KTF exhibiting the highest intensity consistent with experimental expectations. The results demonstrate that while phosphite reduction passivation treatment improves water resistance to some extent in KTFM-P samples, their long-term stability under humid conditions remains inadequate. In contrast, the ethanol-induced deposition-optimized reduction strategy for KTFM-P@KTF phosphors significantly enhanced the phosphors’ long-term water resistance.

To evaluate the stability of phosphors under harsh conditions, boiling tests were performed. Figure 4c illustrates the IQY variations in the three samples before and after boiling treatment. Before boiling, the initial IQY values of KTFM, KTFM-P, and KTFM-P@KTF are 90.2%, 91.1%, and 89.0%, respectively. The slightly higher IQY of KTFM-P is ascribed to the reduced Mn^4+^ content, which alleviates the concentration quenching effect. In comparison, KTFM-P@KTF shows a minor IQY decrease, which arises from the slight luminescence attenuation induced by the relatively thick KTF shell layer formed during ethanol-assisted deposition; this phenomenon has also been documented by Zhou et al. in their research on superhydrophobic KTFM phosphors [14]. After 10 min of boiling, the IQY values of bare KTFM and KTFM-P drop drastically by 87.1% and 51.4%, respectively. In sharp contrast, KTFM-P@KTF retains excellent luminescence properties with only a 12.3% IQY reduction. These results clearly confirm that the KTFM-P@KTF phosphor prepared via ethanol-induced deposition and optimized reduction strategy possesses outstanding structural and luminescent stability under extreme high-temperature and high-humidity environments.

### 3.3. Moisture Resistance Mechanism


(1) $$K_{2}{Ti}_{1-x}{Mn}_{x}F_{6}\left(s\right)\to \left(1-x\right){\left[TiF_{6}\right]}^{2-}(aq)+2K^{+}(aq)+x{\left[MnF_{6}\right]}^{2-}(aq)$$

(2) $${\left[MnF_{6}\right]}^{2-}(aq)+{PO_{3}}^{3-}(aq)+H_{2}O\to {Mn}^{2+}(aq)+2H^{+}(aq)+6F^{-}(aq)+{PO_{4}}^{3-}(aq)$$

The [*MnF*_6_]^2−^ groups on the phosphor surface are prone to hydrolysis, generating insoluble manganese oxides or hydroxides that deteriorate luminescence performance. To address this issue, KTFM phosphors undergo surface passivation treatment using an H_3_PO_3_ aqueous solution. During this process, KTFM partially dissolves and ionizes to release *K*^+^, [*TiF*_6_]^2−^, and [*MnF*_6_]^2−^ groups [21], as illustrated in Reaction (1). Subsequently, *PO*_3_^3−^ undergoes redox reactions with [*MnF*_6_]^2−^ via Reaction (2), which effectively reduces the surface Mn^4+^ content and forms a thin KTF matrix shell with low Mn^4+^ concentration. Nevertheless, the as-formed shell layer remains thin and cannot fully resist hydrolysis under long-term humid conditions. To further improve the water resistance of the material, excess ethanol as an anti-solvent [22,23] is added to make use of residual *K*^+^ and [*TiF*_6_]^2−^ ions in the post-reaction solution and achieve a supersaturated state. Benefiting from the low solubility of K_2_TiF_6_ in ethanol, additional deposition takes place on the initially passivated surface, generating a much thicker KTF matrix shell.

### 3.4. WLEDs Aging Test

**Figure 5:**
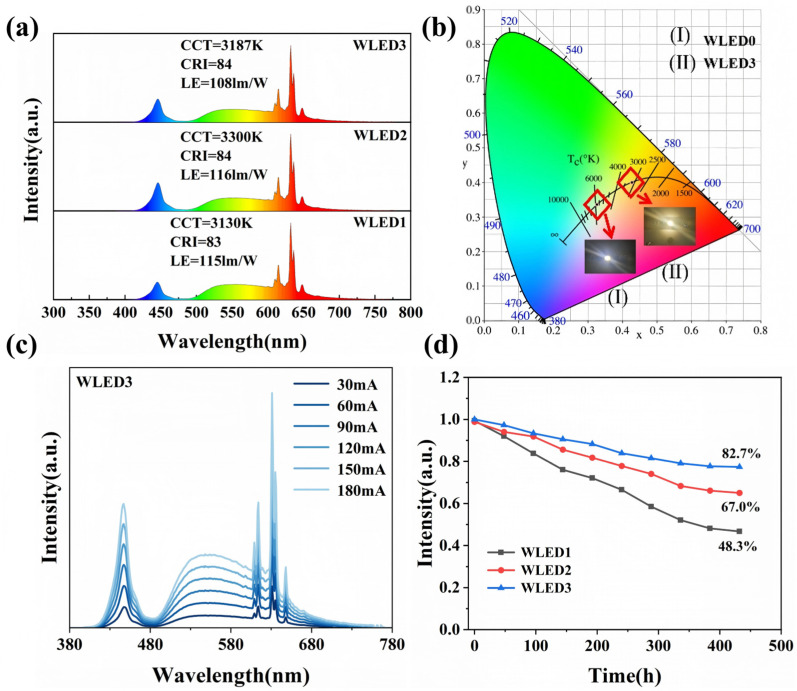
(**a**) EL spectra of WLED1–3; (**b**) International Commission on Illumination (CIE) chromaticity diagrams and working photos of WLED0 and WLED3; (**c**) EL spectra of WLEDs at different driving currents; (**d**) Variation in EL intensity of WLEDs 1–3 with aging time.

The WLED devices fabricated in this experiment utilize blue light chips (~455 nm) as excitation sources, combined with yellow phosphors YAG and red phosphors (KTFM, KTFM-P, KTFM-P@KTF) for encapsulation. Specifically, WLED0 is encapsulated solely with YAG yellow phosphor, while WLED1–3 incorporate KTFM, KTFM-P, and KTFM-P@KTF red phosphors, respectively. Under 100 mA current and 450 nm excitation conditions, the electroluminescence (EL) spectra of each device are measured using an integrating sphere, as shown in Figure 5a. The introduction of red phosphors results in distinct narrow-band sharp emission peaks across the 622–760 nm red light spectrum for WLED1–3, significantly enhancing red light output. Figure 5b shows that WLED0 without red phosphors displays cool white light with CIE color coordinates (0.3265, 0.3366), whereas WLED3 encapsulated with KTFM-P@KTF exhibits warm white light at coordinates (0.4278, 0.4000). Table 2 further lists key performance parameters of each WLED. The correlated color temperature (CCT) of WLED0 is 5772 K, while all WLED1–3 samples with red phosphors exhibit CCT values below 3500 K. In terms of color rendering performance, WLED0 exhibits a color rendering index (CRI) of approximately 56 and a color quality scale (CQS) of 65. In contrast, devices incorporating red phosphors achieve CRI and CQS values exceeding 80 and a luminous efficacy (LE) of 108.8 lm/W, demonstrating a significant improvement in the overall light quality of the white light source. Furthermore, WLEDs encapsulated with modified red phosphors show performance parameters comparable to those of WLEDs using original KTFM materials, indicating that surface modification processes do not adversely affect phosphor emission intensity or overall device performance.

Figure 5c shows the EL spectra of the WLED3 device under varying current ranges from 30 to 180 mA. As the current gradually increases, the shape and position of the luminescence peak remain unchanged, with only the peak intensity exhibiting a corresponding enhancement. This indicates that the encapsulated red phosphor maintains stable light coloration across different current conditions, while the WLED device ensures stable operation under varying current driving ranges within a defined operational scope. In practical applications, WLED devices often require prolonged operation under harsh environments, necessitating validation of their stability under extreme conditions [24,25]. Aging tests were conducted (continuous operation at 85 °C and 85% humidity for 432 h); after the tests, WLED3 incorporating the KTFM-P@KTF phosphor maintained 82.7% of its initial luminous intensity, which is significantly higher than that of WLED1 (48.3%) and WLED2 (67.0%), as illustrated in Figure 5d. These results demonstrate that the ethanol-induced deposition-optimized reduction strategy markedly enhances the water resistance of the KTFM phosphor, enabling WLED devices encapsulated with KTFM-P@KTF to achieve superior long-term operational stability in high-temperature and high-humidity environments.

## 4. Conclusions

In conclusion, this work proposes an ethanol-induced deposition-optimized reduction strategy for enhancing the weather resistance of KTFM red phosphors, and the effectiveness of this strategy is systematically verified through a series of characterizations and performance tests. The strategy involves two key steps: first, phosphorous acid is used as a reducing agent to passivate the KTFM surface, removing unstable surface Mn^4+^ ions and forming an initial K_2_TiF_6_ matrix protective layer; second, leveraging the low solubility of K_2_TiF_6_ in ethanol, additional deposition is induced on the passivated surface to construct a thicker K_2_TiF_6_ shell. Performance tests demonstrate that the modified KTFM-P@KTF phosphor exhibits significantly improved water resistance compared to unmodified KTFM and KTFM-P samples: after 6 h of immersion, KTFM-P@KTF only shows 11.9% fluorescence intensity attenuation, while unmodified KTFM decays to 14.4% of its initial intensity; after boiling treatment, KTFM-P@KTF maintains 88.1% of its initial IQY, far superior to KTFM (3.1%) and KTFM-P. When encapsulated as the red light component in WLED devices (WLED3), KTFM-P@KTF achieves excellent comprehensive performance, with a CCT of 3188 K, CRI of 85, CQS of 82, and LE of 108.8 lm/W at 100 mA. Moreover, WLED3 maintains 82.7% of its initial luminous intensity after 432 h of continuous operation under high-temperature and high-humidity conditions, indicating exceptional long-term operational stability. Overall, the proposed ethanol-induced deposition-optimized reduction strategy effectively solves the poor water resistance issue of KTFM phosphors, and the modified phosphor exhibits outstanding luminescent and environmental stability, which greatly expands the application scope of such red phosphors in the WLED field.

## Figures and Tables

**Table 1:** Content (atomic %) of major elements on the sample surface.

Sample	C1s	O1s	F1s	K2p	Ti2p
KTFM	33.44	13.87	38.42	9.84	4.43
KTFM-P	32.84	11.05	40.80	10.47	4.84
KTFM-P@KTF	30.36	6.57	45.22	12.53	5.32

**Table 2:** Key Photoelectric Parameters of Encapsulated WLEDs.

WLEDs	LE (lm/W)	CCT (K)	CRI	CQS
WLED0	145.5	5772	56	65
WLED1	115.3	3130	83	83
WLED2	116.1	3300	84	80
WLED3	108.8	3188	85	82

## Data Availability

The original contributions presented in this study are included in the article. Further inquiries can be directed to the corresponding authors.

## Abbreviations

The following abbreviations are used in this manuscript:

WLEDs	white light-emitting diodes
IQY	internal quantum yield
EL	electroluminescence
YAG	Y_3_Al_5_O_12_:Ce^3+^
XRD	X-ray diffraction
SEM	scanning electron microscopy
EDS	energy dispersive X-ray spectroscopy
TEM	transmission electron microscope
XPS	X-ray photoelectric spectrum
PL	photoluminescence
LE	luminous efficacy
CIE	International Commission on Illumination
CCT	correlated color temperature
CRI	color-rendering index
CQS	color quality scale
